#  Spontaneous Intestinal Perforation in a Very Low Birth Weight Infant: Successful Management by Peritoneal Needle Suction

**DOI:** 10.21699/jns.v5i3.331

**Published:** 2016-07-03

**Authors:** Fatma-Zohra Chioukh, Karim Ben Ameur, Rachida Laamiri, Hayet Ben Hmida, Abellatif Nouri, Kamel Monastiri

**Affiliations:** 1 Department of Intensive Care and Neonatal Medicine, Teaching Hospital of Monastir, University of Monastir - Tunisia; 2 Department of Paediatric Surgery, Teaching Hospital of Monastir, University of Monastir – Tunisia

**Dear Sir**

The baby was born at 27 weeks gestation to a 31-year-old mother by normal vaginal delivery. Birth weight was 1370 grams and Apgar scores were 9 at one minute and 10 at five minutes. He was admitted to the neonatal intensive care unit for respiratory distress syndrome requiring continuous positive airway pressure (CPAP) support. On the second day of hospital stay, although the general condition of the baby was stable, he developed abdominal distension. However, there was no erythema, tenderness or, a palpable lump. Abdominal X-ray revealed pneumoperitoneum, with free gas under both the domes of diaphragm (Fig.1A). The baby was intubated because his respiratory condition worsened. In view of the stable general condition and without clinical or biological evidence of necrotizing enterocolitis (NEC), and the absence of preceding event which could lead to gastrointestinal perforations, the diagnosis of spontaneous intestinal perforations (SIP) was made. It was decided to do peritoneal needle suction (PNS) at the bedside. One hundred and sixty milliliters of air were removed. The abdominal distension resolved immediately and the control abdominal radiograph showed total resolution of pneumoperitoneum (Fig. 1B). A trial feed was well tolerated and complete enteral feeding was attained 20 days after the PNS. The neonate was discharged 8 weeks later and he is doing well on follow-up. 

**Figure:**
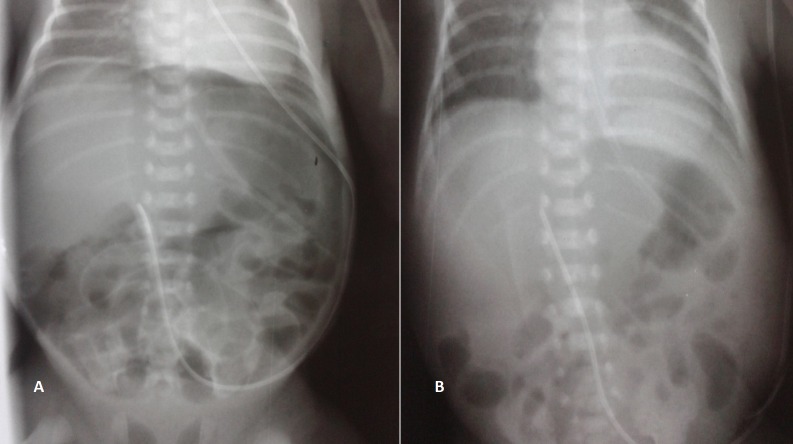
Figure 1: A- Pneumoperitoneum. B- Resolution of pneumoperitoneum after peritoneal needle suction.

In this index case, the etiology of pneumoperitoneum was not evident. It could be the result of nasal ventilation, mechanical ventilation, NEC, or spontaneous. There were no indications for immediate surgical intervention as despite pneumoperitoneum the patient was clinically stable and no features of peritonitis, pain or cardiovascular instability were present. In some patients with clinical evidence of perforation and pneumoperitoneum on x-ray, careful intraoperative search did not reveal any perforation points; instead only evidence of healed perforation was seen.[1] The possibility of spontaneous healing of gut perforations in neonates has been documented and has led to some authors advocating initial conservative management for intestinal perforation.[2] Bedside peritoneal drainage (PD) performed under local anesthesia was first reported in 1977.[3] PD has been utilized as an initial stabilizing procedure and, even as definitive treatment, becoming an alternative to laparotomy in premature neonates with NEC and SIP.[4,5] This minimally invasive procedure can be utilized for unexplained pneumoperitoneum in premature low birthweight neonates not fit for extensive interventions owing to prematurity.

## Footnotes

**Source of Support:** Nil

**Conflict of Interest:** None
